# Health Belief Model, Theory of Planned Behavior, or Psychological Antecedents: What Predicts COVID-19 Vaccine Hesitancy Better Among the Bangladeshi Adults?

**DOI:** 10.3389/fpubh.2021.711066

**Published:** 2021-08-16

**Authors:** Mohammad Bellal Hossain, Md. Zakiul Alam, Md. Syful Islam, Shafayat Sultan, Md. Mahir Faysal, Sharmin Rima, Md. Anwer Hossain, Abdullah Al Mamun

**Affiliations:** ^1^Department of Population Sciences, University of Dhaka, Dhaka, Bangladesh; ^2^Department of Population Science, Jatiya Kabi Kazi Nazrul Islam University, Mymensingh, Bangladesh; ^3^Ovibashi Karmi Unnayan Program, Dhaka, Bangladesh

**Keywords:** health belief model, theory of planned behavior, psychological antecedents, COVID-19, vaccine hesitancy, Bangladesh

## Abstract

This study aimed to determine the prevalence and investigate the constellations of psychological determinants of the COVID-19 vaccine hesitancy among the Bangladeshi adult population utilizing the health belief model-HBM (perceived susceptibility to and severity of COVID-19, perceived benefits of and barriers to COVID-19 vaccination, and cues to action), the theory of planned behavior-TPB (attitude toward COVID-19 vaccine, subjective norm, perceived behavioral control, and anticipated regret), and the 5C psychological antecedents (confidence, constraints, complacency, calculation, and collective responsibility). We compared the predictability of these theoretical frameworks to see which framework explains the highest variance in COVID-19 vaccine hesitancy. This study adopted a cross-sectional research design. We collected data from a nationally representative sample of 1,497 respondents through both online and face-to-face interviews. We employed multiple linear regression analysis to assess the predictability of each model of COVID-19 vaccine hesitancy. We found a 41.1% prevalence of COVID-19 vaccine hesitancy among our study respondents. After controlling the effects of socio-economic, demographic, and other COVID-19 related covariates, we found that the TPB has the highest predictive power (adjusted *R*^2^ = 0.43), followed by the 5C psychological antecedents of vaccination (adjusted *R*^2^ = 0.32) and the HBM (adjusted *R*^2^ = 0.31) in terms of explaining total variance in the COVID-19 vaccine hesitancy among the adults of Bangladesh. This study provides evidence that theoretical frameworks like the HBM, the TPB, and the 5C psychological antecedents can be used to explore the psychological determinants of vaccine hesitancy, where the TPB has the highest predictability. Our findings can be used to design targeted interventions to reduce vaccine hesitancy and increase vaccine uptake to prevent COVID-19.

## Introduction

Though vaccines, in the form of successful mass immunization programs, have saved millions of lives and improved health and wellbeing across the world (1), historically, such successes have constantly been challenged by a minority, yet a significant proportion of vaccine-hesitant individuals and groups for a variety of environmental, cultural, political and psychological reasons (2–5). The World Health Organization has identified vaccine hesitancy as one of the top ten global health threats (6), where vaccine hesitancy has been defined as a delay in acceptance or refusal of vaccination despite its availability (5).

Most studies on vaccine hesitancy come from the Western, educated, industrialized, rich, and democratic (WEIRD) countries (7–9). Thus, very little is known about the contexts of developing, low- and middle-income countries (10). However, the world has witnessed an insurgence of vaccine hesitancy globally during the pandemic of Coronavirus Disease 2019 (COVID-19) (11). A systematic review found that many studies reported a COVID-19 vaccine acceptance rate below 60%, ranging from the lowest 23.6% in Kuwait to the highest 97% in Ecuador (9).

It has been recommended that 60–72% immunity at the population level would be necessary to halt the Coronavirus transmission and community spread (12). The Government of Bangladesh has aimed to achieve an 80% coverage of the COVID-19 vaccination program (13). However, evidence shows that the creation of herd immunity through vaccination is a challenging task when there is vaccine hesitancy (4, 5, 12). Thus, it is crucial to explore vaccine behavior. Unfortunately, there is an apparent dearth of research on COVID-19 vaccination behavior in Bangladesh. The studies conducted in Bangladesh have reported a vaccine hesitancy rate between 29 and 50% (14–17). However, the findings of these studies are not representative of Bangladesh in general as their sample size was small (15, 16), or the data were collected using the online platform (14–16), and there exists a digital divide across the country (18).

Along with quantifying the prevalence of vaccine hesitancy among the population, it is crucial to understand the determinants of the individual decision-making process that result in delay or omission of vaccination (19, 20). Studies found that vaccine hesitancy is predominantly the outcome of the individual decision-making process, which is influenced by individual's feelings about the vaccination or a particular vaccine, barriers, and enablers to vaccinate (10, 19, 20). Thus, it is crucial to understand which psychological drivers determine to delay or refusal of the vaccination (19, 20) so that targeted interventions can be designed to reduce vaccine hesitancy and increase vaccine demand (5, 21).

The health belief model (HBM) is one of the most widely used models in vaccination behavior, particularly in influenza (22), swine flu (23), ebola (22), hepatitis B (24), and COVID-19 (25–27). The key argument of HBM is that the likelihood of an individual adopting a particular health behavior (e.g., getting COVID-19 vaccine) is determined by the perceived susceptibility and severity of illness or disease (e.g., COVID-19), along with the belief in the effectiveness of the recommended health behavior (e.g., COVID-19 vaccination) (28). Thus, the model is comprised of, as applied to COVID-19 and its vaccine, *perceived severity* of *and perceived susceptibility* to COVID-19, *perceived benefits* of and *perceived barriers* to getting a COVID-19 vaccine, and *cues to action* which include implicit or explicit incentives or situations that serve to motivate vaccination, such as information from mass media (25).

In contrast, the theory of planned behavior (TPB) argues that behavior is driven by the intention to carry out the behavior, ultimately determined by an individual's “belief structure” (29). As applied to the context of COVID-19 vaccine, belief structure is comprised of *attitude toward COVID-19 vaccine* (i.e., its perceived necessity, benefit, and effectiveness), *subjective norms* (i.e., whether significant others support getting a COVID-19 vaccine), and *perceived behavioral control* (i.e., to what extent COVID-19 vaccination is perceived within the individual's control) (30). However, new components are continually being added to the TPB framework to increase its usefulness (23). For example, Gallagher and Povey (31) found that the addition of “anticipated regret” substantially increased the predictive value of TPB in terms of older adults' intention to get a seasonal influenza vaccination.

On the other hand, Betsch et al. (10) have incorporated and expanded existing vaccination behavior measures and proposed a framework of 5C psychological antecedents of vaccination. It includes *confidence* (trust in vaccine effectiveness, safety, and necessity, and the system that delivers it), *complacency* (perceiving the disease as low risk), *constraints* (perceived low vaccine availability, affordability, accessibility, and other barriers to vaccinating), the *calculation* (analyzing pros and cons of vaccination), and *collective responsibility* (willingness to take the vaccine for protecting others via herd immunity). The 5C psychological antecedents have explained a greater extent of vaccination variance than other existing models, though it has not been tested alongside the HBM and the TPB (10).

However, in Bangladesh, the studies conducted to understand vaccine hesitancy-related behavior either did not use any theory (14, 17) or used only the health belief model (15, 16). Therefore, this study aimed to examine the predictability of the HBM, the TPB, and the 5C psychological antecedents to understand vaccine hesitancy, which will ultimately help the Government of Bangladesh design the campaign to reduce hesitancy behavior and increase the uptake of the COVID-19 vaccine.

## Materials and Methods

### Study Design and Data Collection

We extracted data for this study from the survey conducted to explore the attitude toward acceptance regarding the COVID-19 vaccine and associated factors among the adults of Bangladesh (32). The original study adopted a cross-sectional research design. The calculated sample size was 1,635, where the Z-score for 95% confidence interval was 1.96, prevalence (p) of COVID-19 vaccine hesitancy from a previous study was 0.325 (17), the margin of error (e) was 0.03, design effect (Deff) for sampling variation was 1.6, and a non-response rate (NR) was 10%. However, 112 respondents did not consent to participate in the survey, while another 26 respondents did not know about the COVID-19 vaccine. Thus, after excluding them, the final sample stood at 1,497 for the analysis.

We collected data from all eight administrative divisions of Bangladesh using probability-proportionate to each division's population size. Both online and face-to-face interviews were conducted to collect data. Data were collected using the online platform Google form from one-third of the respondents. A link to the survey questionnaire was created and sent to the prospective respondents via e-mail, WhatsApp, and Facebook messenger. All the respondents to whom the survey link was sent were requested to share the link in their network to reach more people. The research team members circulated the survey link in their respective professional and social networks. The online link was valid for 3 days. After that, the collected data were downloaded, and divisional distribution was assessed. We then collected data for the remaining sample size of each division using face-to-face interviews. For this, we randomly selected two districts from each division. Within each district, the sample was distributed proportionately according to the rural-urban distribution of its population. Then, convenience sampling was adopted to accomplish face-to-face interviews from the population-based households. The graduate and post-graduate level students of the University of Dhaka were recruited and trained to collect the data. We trained the data collectors through the online platform google meet. The training included discussions on how to conduct face-to-face data collection. The data collection tool was validated and finalized through pretest. The pretest was conducted in both online and face-to-face interviews.

The respondent selection criteria for the face-to-face interview were adult people of 18 years and above living in Bangladesh and knowing about the COVID-19 vaccine. In addition, reading and writing ability and using the Internet were added criteria for selecting the online survey respondents. The data are available in the Mendeley open research data repository (33).

### Measures

#### Outcome Variable: COVID-19 Vaccine Hesitancy

While the other studies conducted in Bangladesh measured the COVID-19 vaccine hesitancy using only one item, we used the following two 6-points Likert-type items to measure COVID-19 vaccine hesitancy among the respondents: (a) *If you get the chance of getting a COVID 19 vaccine for free, what will you do?* (with the response of 1 = *Surely, I will take it*; 2 = *Probably I will take it*; 3 = *I will delay taking it*; 4 = *I am not sure what I will do*; 5 = *Probably I will not take it*; 6 = *Surely, I will not take it*), and (b) *If your family or friends think of getting COVID 19 vaccine, what will you do?* (with the response of 1= *Strongly encourage them*; 2 = *Encourage them*; 3 = *Ask them to delay getting the vaccine*; 4 = *I will not say anything about it*; 5 = *Discourage them to take vaccine*; 6 = *Forbid them to take vaccine*). We combined these two items and calculated the level of COVID-19 vaccine hesitancy, where a higher score indicated a higher level of hesitancy toward the COVID-19 vaccine. The reliability analysis of the scale demonstrates an excellent internal consistency (Cronbach Alpha = 0.833).

#### Predictor Variables

##### The HBM Constructs

The HBM constructs consisted of the following components: perceived susceptibility (included two items, α = 0.657), perceived severity (included two items, α = 0.612), perceived benefits (included three items, α = 0.841), perceived barriers (included five items, α = 0.735), and cues to action (Table 1). These items were measured on a 5-point Likert scale (1 = Strongly Disagree to 5 = Strongly Agree). Cues to action included self-reported COVID-19 positive status for self and family members and sources of vaccine-related knowledge (social media/online news portals/blog, and print media).

**Table 1:** Items used to measure HBM, TPB, and 5C psychological antecedents.

**Statements**	**Cronbach α**
**THE HEALTH BELIEF MODEL** (28)	
**Perceived susceptibility**	0.657
I am worried about the likelihood of getting infected by COVID-19	
I am at high risk of COVID-19 because of my health conditions	
**Perceived severity**	0.612
I will be very sick if I get infected by COVID-19	
I am very concerned that I could die from COVID-19	
**Perceived benefits**	0.841
I think vaccination is good because it will make me less worried about COVID-19	
I believe vaccination will decrease my risk of getting infected by COVID-19	
I think the complications of COVID-19 will decrease if I get vaccinated and then get infected with the Coronavirus.	
**Perceived barriers**	0.735
I am worried that the possible side effects of the COVID-19 vaccination would interfere with my usual activities	
I am concerned about the efficacy of the COVID-19 vaccine	
I have a concern that I may receive faulty/fake COVID-19 vaccine	
It concerns me that the development of a COVID-19 vaccine is too rushed to test its safety properly	
I am concerned about the long-term side effects of the COVID-19 vaccination	
**Cues to action**	NA
Respondents got infected with Coronavirus	
A family member got Infected with Coronavirus	
Social media (e.g., Facebook) or online news portals/blogs as a source of knowledge about the	
COVID-19 vaccine	
Printed newspaper as a source of knowledge about COVID-19 vaccine	
**THE THEORY OF PLANNED BEHAVIOR** (29)	
**Attitude toward vaccine**	0.739
I think the COVID-19 vaccine probably will not work	
I do not trust the COVID-19 vaccine	
I think the COVID-19 vaccine is unnecessary	
I think it is not important to get a vaccine to protect people from the COVID-19	
I do not need a COVID-19 vaccine because I am healthy and at low risk for infection	
I do not need a COVID-19 vaccine because even if I get infected, I will not become seriously ill	
**Subjective norm**	NA
I believe my family members will support me to get vaccinated against COVID-19	
**Perceived behavioral control**	NA
If I want, I can register for COVID 19 vaccination	
**Anticipated regret**	NA
If I do not get a COVID-19 vaccine and end up getting Coronavirus, I will regret not getting the vaccination	
**THE 5C PSYCHOLOGICAL ANTECEDENTS OF VACCINATION** (10)	
**Confidence**	0.808
I am completely confident that COVID 19 vaccines are safe	
I am completely confident that COVID 19 vaccines are effective	
Regarding COVID 19 vaccines, I am confident that public authorities decide in the best interest of the community	
**Constraints**	NA
Everyday work stress may prevent me from getting vaccinated	
**Complacency**	0.753
I think it is unnecessary to receive vaccinations as it cannot prevent COVID-19	
I believe my immune system is powerful; it will protect me from COVID-19	
I believe COVID-19 is not much a severe disease that I should get vaccinated against it	
**Calculation**	0.909
When I think about getting vaccinated against COVID 19, I weigh the benefits and risks to make the best decision possible	
When I think about getting vaccinated against COVID 19, I will first consider whether it is effective or not	
Before I get COVID-19 vaccinated, I need to know about this vaccine in details	
**Collective responsibility**	0.612
I will take COVID 19 vaccine because, in that way, I can protect people with a weaker immune system	
I think vaccination against COVID 19 is a collective action to prevent the spread of diseases	

##### The TPB Constructs

The TPB constructs consisted of four domains: attitude toward vaccine (including six items, α = 0.739), subjective norm, perceived behavioral control, and anticipated regret (Table 1). Each item of the four domains was assessed on a 5-point scale (1 = *strongly disagree* to 5 = *strongly agree*). In addition, the mean score of the items under attitude toward vaccine component was calculated, with a higher average score indicating more negative attitude toward COVID-19 vaccine.

##### The 5C Psychological Antecedents of Vaccination

The 5C psychological antecedents of vaccination consisted of 5 antecedents: confidence (included three items, α = 0.808), complacency (included three items, α = 0.753), constraints (included a single item), the calculation (included three items, α = 0.909), and collective responsibility (included two items, α = 0.612) (Table 1). Each item of the five antecedent domains was assessed on a 5-point scale (1 = *strongly disagree;* 5 = *strongly agree*). Except for the constraints domain, mean scores of items under each domain were computed, with a higher average score indicating the corresponding domain's stronger agreement.

### Other Covariates

We also collected data on the following independent variables: age, sex, religion, marital status, educational attainment, place of residence, geographic region, occupation, number of family members, household income, knowledge about COVID-19 vaccine, knowledge about vaccination process, and behavioral practice to prevent COVID-19.

#### Knowledge About the COVID-19 Vaccine

We assessed the knowledge related to the COVID-19 vaccine using four Likert-type items (32). The total score of these items ranged between 4 and 20, with a higher score indicating higher knowledge. Reliability analysis of the scale suggested an acceptable internal consistency (Cronbach alpha, α = 0.643).

#### Knowledge About the Vaccination Process

Knowledge about the COVID-19 vaccination process was measured using the six binary responses (yes = 1, no = 0) questions (32). The reliability analysis showed good internal consistency among these six questions (α = 0.765). The total score ranged between 0 and 6, with a higher score indicating better knowledge about the COVID-19 vaccination process.

#### Behavioral Practice to Prevent COVID-19

The level of preventive behavioral practices related to COVID-19 was measured using three Likert-type items (32). The total score ranged between 3 and 12, with a higher score indicating a higher level of preventive practices. The reliability analysis showed good internal consistency in this scale (α = 0.857).

### Statistical Analysis

We employed multiple linear regression analysis to assess the selected model's predictability to COVID-19 vaccine hesitancy after checking the assumptions and multi-collinearity. We produced three models. In each model, significant demographic, socio-economic, knowledge, and practice of COVID-19 related variables were controlled to predict the used model. Akaike Information Criterion (AIC), Amemiya Prediction Criterion (APC), Mallows's Prediction Criterion (MPC), and Schwarz Bayesian Criterion (SBC) were used to assess model performance.

### Ethical Approval

The National Research Ethics Committee of the Bangladesh Medical Research Council (BMRC) approved this research (Registration No. 39131012021). The study was carried out following the Declaration of Helsinki. The respondents were informed about the aims, objectives, potential scopes, and implications of this study's findings. Participation in this study was completely voluntary, and no incentive was provided to the participants.

## Results

### Background Characteristics of the Respondents

The demographic, socio-economic, and other background characteristics of the respondents are summarized in Table 2. The respondents' average age was 33.7 years, with a standard deviation (SD) of 12.9. The highest proportion (28.9%) of respondents was from 18–24 years. About 47% of the respondents were women, while most respondents (86.9%) were Muslim. About two-thirds of the respondents (61.6%) were married, while 20.6% of the respondents had less than a secondary education level. About two-thirds of the respondents (64.3%) were from rural areas, while 31.9% were from Dhaka Division. One-third (31.6%) of the respondents were students and unemployed. The mean number of household members was 4.9, while the mean household income was BDT 37627 (Table 2).

**Table 2:** Sample characteristics of the respondents.

**Variables**	**Estimates, *n* (%)**
Age in years	
18–24	432 (28.9)
25–30	362 (24.2)
31–39	254 (17.0)
40–49	236 (15.8)
50+	213 (14.2)
Average age, mean (SD)	33.7 (12.9)
Sex	
Women	692 (46.2)
Men	805 (53.8)
Religion	
Others	196 (13.1)
Muslim	1,301 (86.9)
Marital status	
Never-married	575 (38.4)
Ever-married	922 (61.6)
Education	
No education	129 (8.6)
Primary	179 (12.0)
Secondary and higher secondary	448 (29.9)
Graduate	400 (26.7)
Masters and MPhil/PhD	341 (22.8)
Place of residence	
Rural area	963 (64.3)
Urban area (other than city corporation)	179 (12.0)
City Corporation	355 (23.7)
Administrative division	
Barishal	114 (7.6)
Chattogram	253 (16.9)
Dhaka	478 (31.9)
Khulna	137 (9.2)
Mymensingh	108 (7.2)
Rajshahi	180 (12.0)
Rangpur	114 (7.6)
Sylhet	113 (7.5)
Occupation	
Government, private, and NGO sector job	202 (13.5)
Professional (teacher, engineer, lawyer, doctor, nurse, paramedics, pharmacist)	277 (18.5)
Housewife	348 (23.2)
Students and unemployed	473 (31.6)
Agriculture and day Laborer	102 (6.8)
Others	95 (6.3)
Household members, mean (SD)	4.9 (1.98)
Household income in BDT, mean (SD)	37,627.2
	(81,295.9)
Knowledge about COVID-19 vaccine, mean (SD)	11.4 (2.2)
Knowledge about vaccination process, mean (SD)	2.8 (2.0)
Behavioral practice to prevent COVID-19, Mean (SD)	8.8 (2.7)
Total	1,497 (100.0)

### Predictors of COVID-19 Vaccine Hesitancy in Bangladesh

The total score of the vaccine hesitancy scale ranges from 2 to 12. The mean score of the hesitancy scale was 4.93 (95% CI: 4.79–5.07) with an SD of 2.68. Thus, the accuracy of the hesitancy scale was 41.1% (4.93/12^*^100), indicating that 41.1% (95% CI: 39.9–42.2%) of the respondents had hesitancy to accept the COVID-19 vaccine. To examine the predictability of the HBM, the TPB, and the 5C psychological antecedents, we controlled the effects of demographic, socio-economic, and other COVID-19 related covariates in explaining vaccine hesitancy. Our first model shows that the HBM constructs explained 31% of the variance in COVID-19 vaccine hesitancy (adjusted *R*^2^ = 0.31) (Table 3). Among the HBM constructs, perceived susceptibility, perceived severity, perceived benefits, and perceived barriers were the significant predictors of COVID-19 vaccine hesitancy. An increase in perceived susceptibility of COVID-19 tended to reduce vaccine hesitancy (β = −0.06, *p* < 0.05). Similarly, increased perceived severity of COVID-19 also reduced vaccine hesitancy (β = −0.11, *p* < 0.01). However, among the HBM constructs, perceived benefits had the largest standardized co-efficient. In other words, a one-unit increase in perceived benefits of getting the COVID-19 vaccine reduced the vaccine hesitancy by 0.33 units (β = −0.33, *p* < 0.01). On the other hand, perceived barriers to getting the COVID-19 vaccine tended to increase vaccine hesitancy (β = 0.30, *p* < 0.01). However, the cues to action construct were found insignificant in predicting COVID-19 vaccine hesitancy, except the component “Social media (e.g., Facebook) or online news portals/blog as a source of knowledge about the COVID-19 vaccine.” In other words, respondents who heard about the COVID-19 vaccine from social media (e.g., Facebook) or online news portals were less vaccine-hesitant (β = −0.05, *p* < 0.05).

**Table 3:** Multiple linear regression analysis to predict COVID-19 vaccine hesitancy.

**Variables**	**Adjusted *R*^**2**^**	**Beta (SE)**
**Model 1: Constant + Health belief model constructs (** ***n*** **= 1,497)**	**0.31**	
Perceived susceptibility		−0.06 (0.04)^**^
Perceived severity		−0.11 (0.04)^***^
Perceived benefits		−0.33 (0.03)^***^
Perceived barriers		0.30 (0.02)^***^
Cues to action		
Respondents got infected with Coronavirus		
Yes		−0.03 (0.27)
No (RC)		
A family member got Infected with Coronavirus		
No		0.04 (0.21)^*^
Yes (RC)		
Social media (e.g., Facebook) or online news portals/blogs as a source of knowledge about the COVID-19 vaccine		
Yes		−0.05 (0.14)^**^
No (RC)		
Printed newspaper as a source of knowledge about the COVID-19 vaccine		
Yes		0.01 (0.15)
No (RC)		
**Model 2: Constant + Theory of planned behavior constructs (** ***n*** **= 1,497)**	**0.43**	
Attitude toward COVID-19 vaccine		0.27 (0.02)^***^
Subjective norm		−0.31 (0.07)^***^
Perceived behavioral control		−0.05 (0.05)^**^
Anticipated regret		−0.18 (0.05)^***^
**Model 3: Constant + 5C model constructs (** ***n*** **= 1,497)**	**0.32**	
Confidence		−0.38 (0.03)^***^
Constraints		0.03 (0.06)
Complacency		0.18 (0.03)^***^
Calculation		0.09 (0.03)^***^
Collective responsibility		−0.06 (0.05)^**^

*The effects of the following variables were controlled for all models: sex of the respondents, religion, place of residence, administrative division, knowledge about COVID-19 vaccine, knowledge about vaccination process, and behavioral practice to prevent COVID-19;*

* *indicates p-value < 0.10*,

** *indicates p-value < 0.05*,

*** *indicates p-value < 0.01; RC, Reference category*.

The second model shows that 43% of the variance in COVID-19 vaccine hesitancy was explained by the TPB (adjusted *R*^2^ = 0.43) (Table 3). All four components of the TPB were significant predictors of COVID-19 vaccine hesitancy. Respondents who had a more negative attitude toward the COVID-19 vaccine were more vaccine-hesitant (β = 0.27, *p* < 0.01). Vaccine hesitancy tended to decrease with the increase of familial support in favor of vaccination regarding the TPB's subjective norm (β = −0.31, *p* < 0.01). However, in terms of perceived behavioral control, respondents who mentioned registering for COVID-19 vaccination was within their control were less vaccine-hesitant (β = −0.05, *p* < 0.05). Finally, an increase in anticipated regret among the respondents reduced vaccine hesitancy (β = −0.18, *p* < 0.01).

The third model included the 5C psychological antecedents, which explained 32% of the variance in COVID-19 vaccine hesitancy (adjusted *R*^2^ = 0.32) (Table 3). An increase in COVID-19 vaccine confidence tended to decrease vaccine hesitancy (β = −0.38, *p* < 0.05). The more complacent individuals had a more vaccine hesitancy (β = 0.18, *p* < 0.01). Similarly, respondents who were more calculative about the pros and cons of getting vaccinated or needed more information about the vaccine before getting vaccinated were significantly more COVID-19 vaccine-hesitant (β = 0.09, *p* < 0.01). Finally, respondents who had a sense of collective responsibility to vaccinate against COVID-19 were significantly less vaccine-hesitant (β = −0.06, *p* < 0.05).

Table 4 presents the model summary and the selection criteria to assess model performance. For each of the three multiple linear regression models to identify psychological determinants of COVID-19 vaccine hesitancy, we compared AIC, APC, MPC, SBC to select the best fitting model. The model with the smallest AIC, APC, MPC, SBC values was the best-fitted model. According to these indicators, the TPB constructs (Model 2) were the best-fitted model (Table 4).

**Table 4:** Model summary and selection criteria.

**Model summary**	**Model 1: HBM**	**Model 2: TPB**	**Model 3: 5C**
*n*	1,497	1,497	1,497
*R*	0.56	0.66	0.57
*R* ^2^	0.32	0.43	0.33
Adjusted *R*^2^	0.31	0.43	0.32
F change	108.2	205.09	92.69
df	4, 1,474	4, 1478	5, 1,477
Significance of F change	<0.001	<0.001	<0.001
**Selection criteria**			
Akaike Information Criterion (AIC)	2427.1	2144.6	2399.0
Amemiya Prediction Criterion (APC)	0.70	0.58	0.69
Mallows' Prediction Criterion (MPC)	23.0	19.0	20.0
Schwarz Bayesian Criterion (SBC)	2549.2	2245.5	2505.23

## Discussion

Given the dearth of research on COVID-19 vaccination behavior in Bangladesh, this study aimed to determine the prevalence of COVID-19 vaccine hesitancy and explore its psychological determinants among Bangladeshi adults. To fulfill that intent, we have utilized three of the most widely used tools in the field of vaccination behavior- the HBM, the TPB, and the 5C psychological antecedents of vaccination, and compared their predictability in terms of predicting the COVID-19 vaccine hesitancy. As a result, we found a 41.1% prevalence of the COVID-19 vaccine hesitancy among our study respondents, a significantly higher estimate than found by Kabir et al. (31%) (15), Ali and Hossain (32.5%) (17), and Mahmud et al. (38.8%) (16). This higher estimation may, in part, be explained by the fact that the existing studies (15–17) conducted a rapid assessment of the situation, which may suffer from respondent selection bias. The existing studies also had a small sample size and conducted an online survey (15, 16). In contrast, data were collected through online and face-to-face interviews from a nationally representative sample covering all eight administrative divisions in our study. In that sense, our study findings provide a more accurate estimate of COVID-19 vaccine hesitancy that is generalizable in the context of the adult population living in Bangladesh.

We found that after controlling the effects of socio-economic and demographic variables, level of knowledge related to COVID-19, its vaccine and vaccination process, and level of preventive practices toward COVID-19, the TPB has the highest predictive power (adjusted *R*^2^ = 0.43), followed by the 5C model (adjusted *R*^2^ = 0.32) and the HBM (adjusted *R*^2^ = 0.31) in terms of explaining total variance in COVID-19 vaccine hesitancy among the adults of Bangladesh. This finding is particularly unique because, to date, no study compared the predictability of these three behavioral frameworks or models in terms of predicting vaccine hesitancy, especially in Bangladesh. Moreover, given the newness of the 5C scale (10), the available literature is still scanty that tests this model's predictive validity (34, 35). However, various other studies that adopted only the HBM and the TPB validate our findings that TPB constructs are a better predictor of vaccination behavior (36), specifically in the intention to vaccinate against swine flu (23) human papillomavirus (37) or in the context of COVID-19 vaccination (25, 26, 30).

According to the findings of the HBM constructs, an increase in perceived benefits of the COVID-19 vaccine, along with increasing perceived severity of and perceived susceptibility to COVID-19, significantly reduced the vaccine hesitancy. On the other hand, an increase in perceived barriers to getting vaccinated acted as a significant vaccine hesitancy promoter. These findings essentially correspond to other studies related to influenza vaccination (38), though Lin et al. (27) found perceived susceptibility was not a significant predictor of the COVID-19 vaccine hesitancy in China. In terms of cues to action, though COVID-19 infection status (of both self and family members) were non-significant predictors at a 5% level of significance. We found that social media (e.g., Facebook) or online news portals as the source of information about the COVID-19 vaccine was the significant predictor, and respondents who heard about the COVID-19 vaccine from social media (e.g., Facebook) or online news portals or blogs were less hesitant. Taken together, these findings suggest that imparting adequate and proper information about the COVID-19 vaccine to the public, along with solid evidence of the safety, efficacy, and benefits of the COVID-19 vaccine, can be a crucial strategy to reduce vaccine hesitancy and increase its demand and actual uptake. In that case, social media and online news portals may act as more effective means than printed newspapers to disseminate COVID-19 related information, as found in our study.

According to the findings of the TPB constructs, an increase of negative attitude toward the COVID-19 vaccine and a decrease in perceived behavioral control significantly increased the COVID-19 vaccine hesitancy. Other studies support these findings (25, 36), though perceived behavioral control was found non-significant predictor in other contexts (23, 39, 40). However, subjective norms in family members' support for having COVID-19 vaccination significantly reduced vaccine hesitancy, corresponding to other studies (25, 36). Consistent with earlier research (23, 31, 41), anticipated regret was a significant predictor of COVID-19 vaccine hesitancy. This finding implies that an intervention to increase the COVID-19 vaccination uptake should circulate the message that it is better to get vaccinated than regret later. In addition, alternative measures should be devised to reduce the barriers related to COVID-19 vaccination, such as online registration to get the COVID-19 vaccine. This provision is inconvenient, especially for older persons, people living in rural areas, and those who do not have internet access (42).

Finally, according to the 5C model, more substantial confidence and higher collective responsibility were significantly associated with reduced COVID-19 vaccine hesitancy, whereas increased complacency and calculation significantly increased vaccine hesitancy. These findings are supported by other studies in COVID-19 and other contexts (34, 35), though calculation and constraints were non-significant predictors of COVID-19 vaccine hesitancy among the nurses in Hong Kong (35). In our study, the constraint related to the COVID-19 vaccination was a non-significant predictor of COVID-19 vaccine hesitancy. These findings suggest that public confidence in the vaccine and the health system that delivers the vaccination service are crucial. Widespread misinformation, conspiracy beliefs, and superstitions regarding the COVID-19 vaccine and its potential health hazards have been found to diminish public trust (43) that need to be addressed through proper communication. Extensive information searching about the subjective utility of vaccination, as evidenced in earlier studies (19, 20, 44), might have resulted in more vaccine hesitancy as more calculation increased COVID-19 vaccine hesitancy among the respondents, along with complacency. These findings warrant the urgency of re-iterating the risk communication and health benefits message of getting vaccinated to mass people.

### Strengths and Limitations of the Study

This study is the first to explore the COVID-19 vaccine hesitancy among Bangladeshi adults, adopting the largest and most diversified representative sample. Notably, while the other studies in the context of Bangladesh measured COVID-19 vaccine hesitancy using a single question/item (14–17), we measured it using two items: one item to capture the hesitancy of the respondents for herself/himself, and another one reflected the secondary hesitancy when asked about respondents' opinion about their family members or relatives' hypothetical vaccination decisions. In that way, our study provides a more comprehensive measure of vaccine hesitancy that can be adopted in future research in this field. Moreover, this study also explored many psychological antecedents of the COVID-19 vaccine hesitancy utilizing three of the most widely used theoretical tools- the HBM, the TPB, and the 5C psychological antecedents, and used multivariate modeling to identify the most salient predictors. Therefore, this study's findings can help design targeted interventions to reduce the COVID-19 vaccine hesitancy, which will help the Government of Bangladesh attain the target of 80% vaccination coverage for the COVID-19 vaccine. Another strength of the study is that our study provides the findings on the prevalence of the COVID-19 vaccine hesitancy and its predictors while the COVID-19 vaccine was publicly available in Bangladesh. Finally, in terms of theoretical contribution of the study in the field of vaccination behavior, this study contributes evidence from a non-WEIRD country that, at one hand, assess the predictive validity of the 5C psychological antecedents of vaccination (10) and, on the other hand, validates the theoretical supremacy of the TPB over the HBM and the 5C model in predicting the COVID-19 vaccine hesitancy among the adult population in Bangladesh. However, our study also has some limitations. This study could not use probability sampling completely. We tried to draw our sample following the national population distribution regarding age, sex, residence, region, marital status, and religion. However, the distribution of education among the respondents is not comparable to national data. Moreover, this study collected self-reported data that may suffer from reporting bias. Finally, this research used a cross-sectional study design which cannot establish causality.

## Conclusion

This study provides evidence that theoretical frameworks like the TPB, the HBM, and the 5C psychological antecedents can explore the psychological determinants that influence a person's vaccination decision-making process. Among the frameworks of determinants, the TPB has the highest predictive power in determining the vaccination decision. These findings can be used to craft targeted interventions to reduce vaccine hesitancy and increase vaccine uptake. Thus, this study's findings will steer Bangladesh's vaccination campaign and those alike to reach the targeted coverage of the COVID-19 vaccination program and, thereby, paving the way for successful prevention of the never-ending pandemic COVID-19.

## Data Availability Statement

The dataset presented in this study can be found at https://doi.org/10.17632/jzvbvvknkv.1.

## Ethics Statement

The studies involving human participants were reviewed and approved by the National Research Ethics Committee of the Bangladesh Medical Research Council (BMRC). The patients/participants provided their written informed consent to participate in this study.

## Author Contributions

MBH conceptualized the study. MBH, MAH, and MZA analyzed and interpreted the data and drafted the manuscript. MSI, SS, MMF, SR, and AAM revised the manuscript critically for valuable intellectual content and approved the final version to be published. All the authors designed the study, collected the data, and remained in agreement to be accountable for all aspects of the work.

## Conflict of Interest

The authors declare that the research was conducted in the absence of any commercial or financial relationships that could be construed as a potential conflict of interest.

## Publisher's Note

All claims expressed in this article are solely those of the authors and do not necessarily represent those of their affiliated organizations, or those of the publisher, the editors and the reviewers. Any product that may be evaluated in this article, or claim that may be made by its manufacturer, is not guaranteed or endorsed by the publisher.
